# Erythropoietin: A Potent Inducer of Peripheral Immuno/Inflammatory Modulation in Autoimmune EAE

**DOI:** 10.1371/journal.pone.0001924

**Published:** 2008-04-02

**Authors:** RuiRong Yuan, Yasuhiro Maeda, Weiping Li, Wei Lu, Stuart Cook, Peter Dowling

**Affiliations:** 1 Department of Veterans Affairs, New Jersey Health Care System, East Orange, New Jersey, United States of America; 2 Department of Neurosciences and Neurology, UMDNJ-New Jersey Medical School, Newark, New Jersey, United States of America; Centre de Recherche Public-Santé, Luxembourg

## Abstract

**Background:**

Beneficial effects of short-term erythropoietin (EPO) therapy have been demonstrated in several animal models of acute neurologic injury, including experimental autoimmune encephalomyelitis (EAE)-the animal model of multiple sclerosis. We have found that EPO treatment substantially reduces the acute clinical paralysis seen in EAE mice and this improvement is accompanied by a large reduction in the mononuclear cell infiltration and downregulation of glial MHC class II expression within the inflamed CNS. Other reports have recently indicated that peripherally generated anti-inflammatory CD4^+^Foxp3^+^ regulatory T cells (Tregs) and the IL17-producing CD4+ T helper cell (Th17) subpopulations play key antagonistic roles in EAE pathogenesis. However, no information regarding the effects of EPO therapy on the behavior of the general mononuclear-lymphocyte population, Tregs or Th17 cells in EAE has emerged.

**Methods and Findings:**

We first determined in vivo that EPO therapy markedly suppressed MOG specific T cell proliferation and sharply reduced the number of reactive dendritic cells (CD11c positive) in EAE lymph nodes during both inductive and later symptomatic phases of MOG_35–55_ induced EAE. We then determined the effect in vivo of EPO on numbers of peripheral Treg cells and Th17 cells. We found that EPO treatment modulated immune balance in both the periphery and the inflamed spinal cord by promoting a large expansion in Treg cells, inhibiting Th17 polarization and abrogating proliferation of the antigen presenting dendritic cell population. Finally we utilized tissue culture assays to show that exposure to EPO in vitro similarly downregulated MOG-specific T cell proliferation and also greatly suppressed T cell production of pro-inflammatory cytokines.

**Conclusions:**

Taken together, our findings reveal an important new locus whereby EPO induces substantial long-term tissue protection in the host through signaling to several critical subsets of immune cells that reside in the peripheral lymphatic system.

## Introduction

Most autoreactive T cells are negatively selected and eliminated during thymic development in a process called central T cell tolerance [1], [2]. However, the central selection process is often incomplete and autoreactive lymphocytes with pathogenic potential still circulate in the peripheral lymphoid tissues [3], [4]. When abnormally activated by “self” antigens or mimics, these autoreactive T cells may attack self-organs thereby participating in the development of autoimmune disorders. This fact is supported by the demonstrable ability to induce a variety of autoimmune diseases in rodents using appropriate immunization protocols. Th17 cells are a unique subset of CD4^+^ T_H_ cells and recent studies suggest that Th17 cells have an important pathogenic role in T cell mediated autoimmune disease and tissue inflammation [5], [6], [7], [8], [9]. Another distinct T cell population (CD4^+^Foxp3^+^) called regulatory T cells (Tregs) has also attracted attention recently because it plays an important role in maintenance of peripheral tolerance and in controlling the destructive self-reactive T cells found in autoimmune animal models of arthritis, multiple sclerosis, diabetes, and inflammatory bowel disease [10], [11], [12]. Modulating the Treg population and other associated factors may prove to be an effective new therapy for autoimmune disease.

EAE is a central nervous system inflammatory demyelinating disease of animals that attacks the spinal cord and brain white matter [13], [14]. The CNS white matter histopathology in EAE animals is similar to that in multiple sclerosis (MS) in that there are prominent white matter perivascular infiltrating T cells, myelin destruction and associated axonal damage. Autoreactive CD4 and CD8 T cells are believed to be involved in the pathogenesis of EAE and MS [15], [16], [17], [18]. Subcutaneous injection of myelin antigen into animals or adoptive transfer of purified MOG-specific T cells obtained from EAE mice into naïve mice can both induce the disease [18], [19]. Proinflammatory cytokines, especially IL-2 and TNF-alpha have been demonstrated to play a significant role in both the induction phase and later clinical stages of EAE pathogenesis [20], [21], [22].

Erythropoietin (EPO), a 165 amino acid glycoprotein cytokine, has been used extensively for the treatment of anemia in humans and has been shown to have the capacity to induce neuroprotection in a disparate variety of animal models of nervous system disease [14], [23], [24], [25]. We have previously demonstrated that EPO therapy is of substantial clinical benefit and EPO treatment significantly reduces mononuclear cell infiltration, downregulates MHC class II expression, and induces axonal protection within the brain and spinal cord of animals with EAE [14].

The mechanism by which EPO induces neuroprotection in EAE remains unclear. In this study, we characterized the effect of EPO on immunogenesis within the peripheral lymphoid tissues and the impact of EPO therapy on infiltrating inflammatory cells within the CNS target organ using the EAE mouse model. We found that EPO therapy exerts unexpectedly profound biologic effects within the peripheral lymphoid system tissues and acts as a potent molecular check on inflammation by blocking dendritic cell expansion and T cell proliferation while inducing rapid expansion of the Treg cell population and a corresponding reduction in Th17 cells. This comprehensive immune modulating mechanism likely accounts for the bulk of the EPO neuroprotective effects induced in the EAE animal model.

## Results

### EPO down-regulates MOG-specific T cell proliferation in vitro and suppresses T cell cytokine production

**Figure 1:**
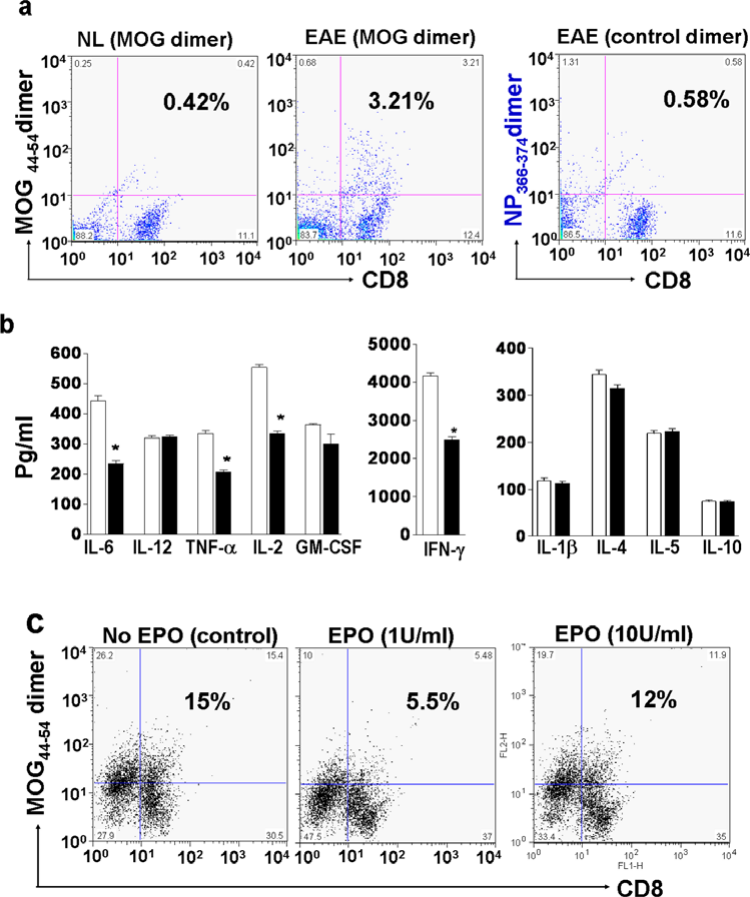
EPO down-regulates proliferation of MOG-specific T cells and suppresses T cell cytokine production. a) Single cell suspensions of hyperimmune EAE spleen were prepared 7 days post MOG peptide immunization and quantified by flow cytometry for CD8 and MOG_44–54_/H-2Db dimer double positive binding. In normal mice, 0.42% of splenic CD8 cells bound to the MOG_44–54_/H-2Db dimer (left side), whereas an 8-fold increase in MOG_44–54_/H-2Db dimer positive staining occurred in CD8 T cells obtained from MOG_35–55_ immunized EAE animals (middle). In contrast, when a non-specific control peptide was tested, only 0.6% of CD8 cells exhibited non-specific binding to the NP_366–374_ dimer (right). b & c) MOG-EAE antigen specific T cells were enriched by exposure to antigen in long term tissue culture, and then exposed to EPO (1 or 10 U/ml) for 72 hrs. Supernatants were collected from the cultured MOG-T cells for cytokine profile analysis and the remaining cells were quantified for number of CD8 and MOG_44–54_/H-2Db dimer double binding cells after exposure to EPO or control treatment. EPO induced a marked decrease in pro-inflammatory cytokine production (black bars) when compared to controls (white bars). EPO at 1 U/ml induced a substantial decline in the MOG-specific CD8 T cell population (c-middle panel). Some of the MOG_44–54_-peptide/H-2Db dimer positive, non-CD8 T cell (including portion of CD11c positive cells) populations were also reduced by EPO treatment (c-middle panel). Each data point is the mean±SEM of three experiments performed in triplicate. *, p<0.005.

Autoreactive T cells are believed to play key roles in the pathogenesis of EAE. Two major pathogenic MOG peptide epitopes (MOG_40–54_ and MOG_44–54_) have been used to construct peptide/H-2D^b^ dimeric complexes to quantify the encephalitogenic CD8 T cell subsets [19]. We first determined if peripheral MOG-specific CD8 T cells in MOG_35–55_ immunized mice could be quantified by flow cytometry. As expected, less than 0.5% of normal mouse spleen mononuclear cells stained positive with the MOG_44–54_/H-2D^b^ dimer (Fig. 1a). By contrast, about 3% of spleen mononuclear cells obtained from MOG_35–55_ peptide immunized mice were double positive for MOG_44–54_/H-2D^b^ dimer and CD8 staining 7 days post immunization (Fig. 1a). In contrast, we found less than 0.6% non-specific binding to a control NP_366–374_/H-2D^b^ dimer by the same EAE splenic single-cell suspensions (Fig. 1a). Adoptive transfer of these EAE-MOG induced T cells generated clinical EAE in naïve normal C57 mice and CNS pathologic changes in recipient mouse spinal cords about 14 days after adoptive transfer (data not shown).

To determine whether EPO exerted a direct effect on MOG-specific T cells and their cytokine profile, we enriched EAE MOG-specific T cells in long term culture and then treated them with EPO (1, 10,100 U/ml) for 48–72 hrs. Culture supernatants were collected for cytokine quantification and the remaining cell population was reacted with peptide/dimer complexes and anti-CD8 antibody to determine the number of MOG specific T cells. EPO (1 U/ml) induced a substantial reduction (*p<0.005) in major pro-inflammatory cytokines (IL-6, TNF-α, IL-2 and IFNγ) (Fig. 1b). A mild reduction in level of GM-CSF (p = 0.09) and insignificant changes in IL-4, IL-10, IL-5 and IL-12 levels were observed (Fig. 1b). We also found a marked reduction in numbers of MOG antigen specific T cells in EPO treated samples (p<0.005) and this correlated well with the marked reduction in multiple pro-inflammatory cytokine levels (Fig. 1c). The EPO-mediated downregulation of MOG-specific T cell proliferation was dose related and the most effective reductions were observed in lower dose EPO treated samples (1 and 10 U/ml), whereas high dose EPO (100 U/ml) in cell culture had little effect on numbers of MOG-T cells and levels of cytokine (data not shown).

### EPO treatment reduces inflammatory mononuclear cell (MNC) numbers in peripheral lymph nodes

**Figure 2:**
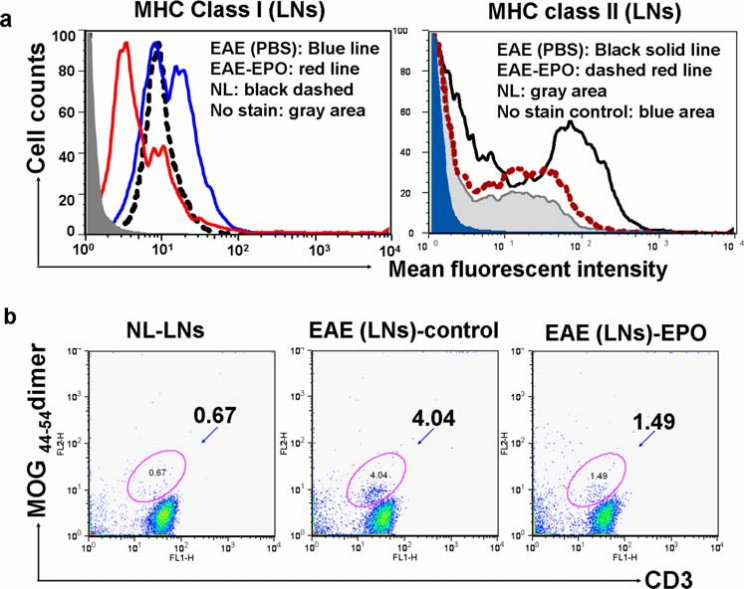
In vivo effect of EPO treatment on MHC expression and number of MOG antigen specific T cells in inguinal lymph nodes. EPO treatment was started 7 days after mice received MOG_35–55_ immunization. Bilateral draining inguinal lymph nodes (DILNs) were obtained from mice on day 11 (after 3 day treatment with either EPO at 5000 U/kg/day or sham treatment with PBS) and single cell suspensions were prepared. a) EPO treatment down-regulated mononuclear cell MHC class I (left) and class II (right) expression in lymph nodes from EAE mice. b) Less than 0.7% of single cells from normal mouse inguinal lymph nodes were double-positive for MOG_44–54_ dimer and anti-CD3 antibody. In contrast, about 4% of single cell suspensions from sham treated EAE mice were positive for FITC-CD3 and PE-MOG_40–54_/H-2Db dimer double staining (middle), whereas EPO treatment reduced in vivo proliferation of MOG-specific T cells back to 1.5% (right).

We previously reported that both disease severity and duration of maximum impairment were reduced when EPO therapy was initiated after onset of neurologic impairment in MOG-EAE mice [14]. Others have reported that early EPO treatment during the EAE induction period (prior to or immediately after MOG_35–55_ immunization) was also effective in delaying disease onset and reducing disease severity [23], [25]. To assess if EPO treatment post MOG-immunization had direct immunomodulating effects within the peripheral lymphoid tissue of EAE mice, EPO or sham PBS treatment was initiated 7 days post immunization and draining inguinal lymph nodes (DILNs) from EPO or sham treated EAE mice were subsequently obtained at different time points. The total number of MNCs, CD4, CD8 T cells and CD11c positive dendritic cells as well as MHC class I and class II expression on the DILN cells was quantified by flow cytometry at different times. Table 1 shows a greatly expanded inflammatory mononuclear cell population occurs in sham treated EAE mice on day 11 post MOG-immunization compared to normal controls and the inflammatory cell subset numbers also remained higher than normal on day 17. EPO treatment for as few as 3 days (day 11 post MOG-immunization) significantly reduced all subsets of the inflammatory cells (p<0.001) and a similar reduction in cell subsets was observed on day 17. We also found that EPO treatment for 3 days down-regulated MHC class I and II expression in DILN mononuclear cells (Fig. 2a, p<0.001). About 4.0% of the spleen mononuclear cells were double positive for CD3 and MOG_44–54_ dimer binding and EPO treatment significantly reduced the MOG-specific T cell population in EAE mice (Fig. 2b, p<0.01).

**Table 1:** EPO reduces total number of Mononuclear and T cells in EAE lymph nodes

	Day 11 a			Day 17 b		
Cells (×10^6^)	Normal	EAE-PBS	EAE-EPO	Normal	EAE-PBS	EAE-EPO
Mononuclear cells (MNCs)	6.15±0.79^c^	37.60±9.34^d^	6.76±1.45	5.30±0.87	23.6±4.78^d^	10.86±3.52
CD4 T cells	2.15±0.17	11.76±2.05^d^	5.58±0.89	2.1±0.56	4.24±0.52^e^	3.34±0.42
CD8 T cells	1.53±1.71	8.4±0.52^d^	1.64±0.18	1.71±0.36	3.66±0.59^e^	2.44±0.21
Dendritic cells (DC)	0.16±0.01	1.59±0.12^d^	0.07±0.01	0.15±0.02	0.7±0.19^d^	0.2±0.01

a, b EPO or sham PBS daily treatment for 3 days was initiated 7 days post MOG-immunization. Mice (n = 6/group) were sacrificed on day 11 (^a^) or day 17 (^b^). DILNs were harvested for cell quantification. Normal mice (n = 6) were used as controls.

c mean±SEM

d p<0.001 (sham PBS treated EAE vs NL or EPO-treated EAE mice)

e p<0.05 (sham PBS treated EAE vs NL mice)

**Figure 3:**
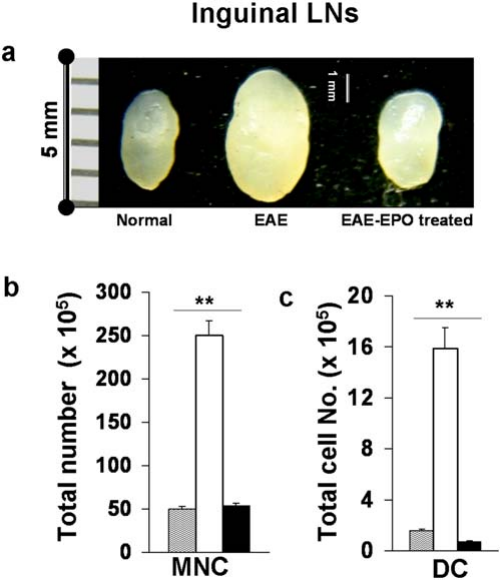
Early EPO treatment sharply reduced total numbers of inflammatory cells and limited expansion of the dendritic cell population in DILNs during the EAE induction phase. C57 mice received EPO or PBS sham treatment starting on the day of MOG-immunization for 6 days and lymph nodes were obtained on day 7. a) Significantly enlarged inguinal lymph nodes were observed in control sham treated MOG-EAE mice (middle), whereas much smaller nodes were found in EPO treated EAE mice (right) similar in size to nodes from normal animals (left). b) Total MNCs were quantified from single cell suspensions of DILNs after lysis of RBCs. Greatly increased inflammatory mononuclear cell numbers were observed in sham treated EAE lymph nodes compared to DILNs from EPO treated EAE mice or normal mice. c) Cells were reacted with fluorophore labeled mAbs specific for surface markers (MHC class II and CD11c) and analyzed by flow cytometry. Note that the large expansion in dendritic cell population found in sham treated EAE mice was dramatically suppressed by EPO therapy. Data represents mean±SEM for 6 individual mice. **, p<0.0001.

We next determined if EPO had similar immunomodulating effects on the peripheral lymphoid tissues when EPO treatment was started at the same time as the mice received MOG peptide immunization. Mice were immunized with MOG_35–55_ and then divided randomly into two groups that received either immediate EPO treatment (5000 U/kg/day ×3 followed by 1000 U/kg/day×3) or PBS sham treatment over the same period for a total of 6 days. DILNs and spleens were collected from mice on day 7. Fig. 3a shows greatly enlarged DILNs were induced in sham treated MOG-EAE by 7 days post immunization. In contrast, the size of DILNs in EPO treated EAE mice was much smaller and resembled lymphoid tissue from normal mice. When total mononuclear cells were quantified from single cell suspensions of harvested inguinal lymph nodes, we again found much higher cell numbers in sham treated EAE mice compared to EPO treated EAE mice or to normal mice (Fig. 3b). EPO therapy significantly reduced the population of CD11c positive DCs (Fig. 3c) and the numbers of CD4, CD8 T cells (data not shown).

### EPO treatment induces peripheral Treg cell expansion and blocks Th17 cell polarization in MOG-EAE mice

**Figure 4:**
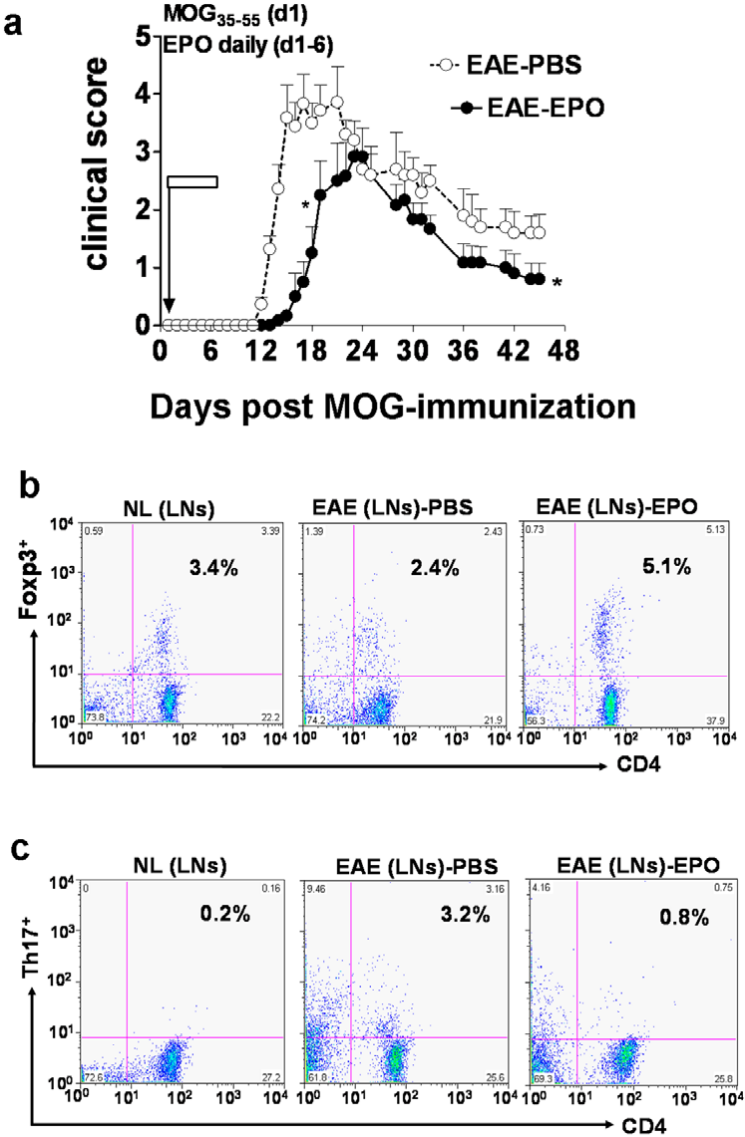
EPO induced sustained immuno/inflammatory modulation by expanding peripheral Treg cell numbers and reducing Th17 positive cells. Mice were immunized with MOG and received daily EPO treatment for 6 days (day1–6). a). EPO therapy for 6 days delayed onset of clinical neurologic signs in animals and reduced the magnitude of clinical deficit in EAE mice (•) compared to sham treated control (○) EAE mice (*, p<0.05). The clinical score was determined as the mean±SEM of 8 mice per group. Data represents the mean±SEM of 8 individual mice. b–c) DILNs were obtained from mice after 6 days treatment with either EPO or PBS. Cells were quantified for number of Tregs (CD4^+^Foxp3^+^) and Th17 by flow cytometry. Panel b shows that about 3.4% Treg cells were detectable in normal mouse inguinal lymph node (left). A reduced number (2.4%) of Treg cells was detected in sham treated EAE control mice nodes (middle), whereas EPO therapy induced a 2-fold increase in Foxp3+ Treg cells on day 16 compared to sham treatment (right). Panel c shows <0.2% of normal healthy lymph node cells stained positive for Th17 (left). Numbers of peripheral Th17 cells greatly increased on day 16 (15-fold) in EAE mice treated with PBS (middle, 3.2%). In marked contrast, EPO therapy (right) sharply reduced the number of peripheral Th17 cells in MOG-immunized animals.

Brief treatment with EPO during the EAE induction period for 6 days significantly delayed onset of clinical neurologic deficit compared to sham treated EAE mice (P<0.05). This brief course of EPO treatment induced a neuroprotective effect that was sustained over a long term clinical follow-up period of 45 days (Fig. 4a). Both Treg and Th17 cells are believed to be critical for control of the autoimmune inflammatory process in EAE and other autoimmune diseases including collagen-induced arthritis and inflammatory bowel disease [26], [27], [28], [29]. We suspected that EPO-mediated neuroprotection in EAE might be in part a consequence of an immunomodulating effect on the balance of Treg and Th17 cell populations in either peripheral lymphoid tissue or the central nervous system target organ. We found a significant reduction in the number of peripheral Treg cells and a remarkable increase in peripheral Th17 cells during the early symptomatic phase (day 16 post MOG-immunization) in sham treated EAE animals compared to normal controls (Fig. 4b–c). EPO treatment for 6 days induced significant expansion in Treg cells within the DILNs (Fig. 4b–c) and significantly reduced the polarization of Th17 cells in the periphery.

**Figure 5:**
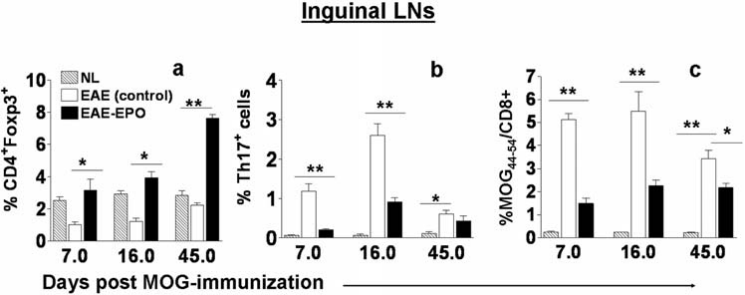
EPO treatment induced peripheral Treg cell expansion while reducing MOG-specific T cells and Th17 positive cells in peripheral lymphoid tissues. Mice were immunized with MOG and received daily EPO treatment for 6 days (day1–6). DILNs and spinal cords were obtained at three different time points (day 7, day 16 and day 45) from mice treated with EPO or sham treated with PBS. Cells were quantified for number of Tregs (CD4^+^Foxp3^+^), Th17 and MOG-antigen specific T cells by flow cytometry. a) There were reduced numbers of Treg cells in DILNs from sham treated EAE mice at early stages (day 7) as well as at the peak of clinical signs (d16), and during recovery (day 45) compared to normal healthy control animals. b) Numbers of peripheral Th17 cells increased in sham treated EAE mice, whereas EPO therapy sharply reduced the number of peripheral Th17 cells in MOG-immunized animals. c) EPO treatment significantly reduced peripheral MOG_40–54_/H-2Db specific T effector cell population compared to sham treated EAE mice. Data represents mean±SEM for 6 individual mice. *, p<0.05; **, p<0.001.

To serially quantify the Tregs, Th17 cells and MOG_40–54_-specific T cells in EAE animals after treatment with either EPO or sham PBS, fresh MNCs from DILNs of MOG-immunized mice were obtained at different time points after initiation of either EPO or sham PBS treatment. We found sustained reductions in Treg cells during the early EAE induction period, the symptomatic phase as well as the late chronic phase in sham treated EAE animals compared to normal controls (p<0.01). The reduction in peripheral Treg cells in sham treated EAE was more apparent on day 7 (before onset of clinical signs) and on day 16 (early acute clinical disease) than later in the disease course. EPO treatment induced a significant expansion in Treg cells within the peripheral lymphoid tissue that could be detected at a very early stage (Fig. 5a). The increased number of peripheral Tregs was even more prominent 5 weeks after termination of EPO treatment (p<0.001). The Th17 cell population in peripheral lymphoid tissue remained elevated in sham treated EAE mice during both the early and late stages of EAE (Fig. 5b), as expected. The Th17 expansion was associated with a fulminant inflammatory response following MOG-immunization in untreated EAE animals and was associated with severe clinical disease. EPO therapy significantly blocked polarization of peripheral Th17 cells (p<0.001). In addition, early EPO treatment again reduced peripheral MOG_40–54_/H-2Db antigen specific T cells while inducing sustained suppression of the MOG-T effector cell population (p<0.001, Fig. 5c). The expansion in peripheral Treg cells, and marked reduction in peripheral Th17 and MOG_40–54_/H-2Db specific T cells as well as the abrogation of dendritic cell proliferation that occurred in EPO treated EAE mice clearly correlated well with less severe clinical disease.

### EPO treatment increased numbers of Treg cells and reduced numbers of Th17 cells in MOG-EAE mouse spinal cord

**Figure 6:**
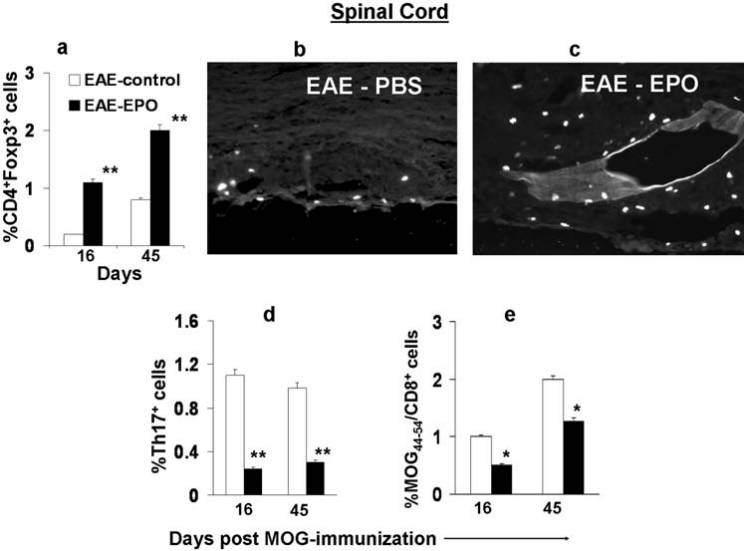
EPO treatment increased numbers of Treg cells and reduced numbers of Th17 cells in MOG-EAE mouse spinal cord. MOG-immunized C57 mice received daily EPO or PBS treatment for 6 days (day 1–6). Spinal cords were obtained at two different time points (day 16 and day 45) from mice treated with EPO or sham treated with PBS. Cells were quantified for number of Tregs (CD4^+^Foxp3^+^), Th17 and MOG-antigen specific T cells by flow cytometry. a) EPO therapy induced a substantial increase in Foxp3+ Treg cells in EAE spinal cords. The EPO induced expansion of Tregs in the CNS became even more evident in late stages of the disease and correlated with less severe neurologic deficit. b–c) Foxp3^+^ labeled Treg cells in PBS or EPO treated MOG-EAE spinal cord by IHC. Panel b shows a sham treated EAE spinal cord section reacted with Foxp3 antibody containing few labeled cells. By contrast, many more Foxp3+ cells were present in the infiltrates of EPO treated EAE spinal cord (panel c). d) Significantly increased numbers of Th17 cells occurred in sham treated EAE mouse spinal cord and this correlated with more severe clinical neurologic deficits whereas EPO therapy suppressed the number of spinal cord Th17 cells. e) Increased MOG antigen-specific T cells occurred within untreated EAE spinal cord, and EPO induced large reductions in MOG-specific T effector cells within the CNS. Data represents mean±SEM for 6 individual mice. *, p<0.05; **, P<0.001.

During the EAE induction phase, we found increased numbers of inflammatory cells within spinal cords shortly before onset of paralysis in sham treated EAE mice (about day 7–10), In contrast far fewer infiltrating inflammatory cells were found in spinal cords from EPO treated EAE mice. The number of CNS infiltrating inflammatory cells was 4–5 times greater in sham treated EAE mice compared to EPO treated animals when animals were studied either at their peak clinical neurologic deficit or at later stages of EAE (day 45). Detailed analysis of the inflammatory cells infiltrating the CNS again revealed higher numbers of Treg cells in EPO treated animals (Fig. 6a). The increased Treg cell population in the diseased CNS similarly became more evident in late stages of the disease, suggesting that Treg cells continue to migrate into the CNS for a lengthy period of time (Fig. 6a). The increased number of anti-inflammatory Treg cells infiltrating the spinal cord in EPO-treated EAE animals was confirmed by direct immunohistochemical staining for Treg cells (mAb against Foxp3^+^) on paraffin-embedded spinal cord sections. Fig. 6 (b–c) shows a substantial increase in infiltrating Treg cells within the spinal cord of EPO-treated EAE (Fig. 6b) compared to sham treated EAE (Fig. 6c).

Significantly increased Th17 cells within the CNS were found at the time animals developed clinical disease, and higher numbers of Th17 cells in spinal cords were notably associated with more severe clinical neurologic deficits in EAE animals. EPO treatment reduced infiltrating Th17 cells in the diseased CNS (Fig. 6d) as previously described in peripheral lymph nodes. Similarly, a marked reduction in MOG-T effector cells within the CNS as judged by the antigen dimer assay was observed in EPO treated animals compared to sham treated EAE controls (Fig. 6e).

## Discussion

EAE is a commonly studied animal model of acute central nervous system inflammatory demyelinating disease, and it is believed that autoreactive T cells and proinflammatory cytokines play key roles in the early inductive phase and later paralytic stages of EAE pathogenesis [16], [30], [31]. We have previously demonstrated that EPO treatment blocks clinical progression and blunts the disease pathology in MOG-induced EAE [14]. In this study, we utilized in vitro and ex vivo methods to explore potential mechanisms by which EPO mediates neuroprotection in the EAE animal model. We found in vitro that exposure of enriched EAE splenic T cell cultures to EPO significantly downregulated autoreactive T cell numbers and their production of proinflammatory cytokines. EPO treatment in EAE mice also greatly reduced total MNC numbers in lymphoid system tissue, including the antigen presenting DC population as well as T cells. Most importantly, EPO treatment also resulted in a marked expansion in the Treg cell population and a corresponding reduction in Th17 cells and MOG-specific T cells within both peripheral lymphoid tissue and diseased EAE spinal cords. By modulating multiple functional immune cell types and their corresponding cytokines, EPO therapy transformed a significantly destabilized autoimmune process in EAE animals into one of relatively normal immune status. As a consequence, less severe neurologic deficits and less neuropathologic abnormalities were found in EPO treated EAE animals compared to sham treated controls.

Traditionally CD4 T cells have been considered to be the critical element in EAE pathogenesis, but recent studies have indicated that CD8 cells may also play an important role in EAE induction and disease progression [16], [17], [18], [30]. Studies on human CNS tissue also support the notion that myelin-specific CD8 T cells in human MS lesions may be encephalitogenic [30], [32]. A truncated MOG peptide library derived from MOG_35–55_ has been previously used to identify the key peptide epitopes for pathogenic T cells and two major MOG peptide epitopes within MOG_40–54_ have been found to be pathogenic for EAE induction [19]. To determine if EPO had a direct impact on MOG-specific T cells and their associated cytokines, we studied MOG-specific T cells in culture after exposure to EPO or medium alone. Low dose EPO significantly reduced the proliferation of MOG_44–54_/H-2D^b^ specific CD8 T cells, while much higher doses (100 U/ml) had only minor or no effect. A similar J-shaped dose-response curve following EPO exposure has been previously observed in MHC class II expression on a mouse EOC microglial cell line [14].

Since proinflammatory cytokines, especially IL-2, IL-6 and TNF-α, play significant roles in EAE pathogenesis [20], [21], [33], we collected supernatants from either EPO or control media treated cell cultures and quantified levels of cytokines. Exposure to EPO induced substantial reductions in all of the major classic pro-inflammatory cytokines and the reduced cytokine level in EPO treated samples was associated with decreased numbers of MOG_44–54_ dimer positive pathogenic T cells. There were no significant alterations in Th2-type cytokines such as IL-4 and IL-5 in EPO treated samples compared to controls. The in vitro EPO-mediated reduction in proinflammatory cytokines was also dose dependent.

The EPO-mediated non-specific and specific immune modulating effects on peripheral lymphoid tissues in EPO-treated EAE animals likely contributes to their less severe clinical course. Anti-inflammatory regulatory T cells have been found to be an important CD4 cell subpopulation for controlling the development of autoimmune disease [12], [34], [35], [36], [37], [38]. Adoptive transfer of Treg cells has been shown to be of therapeutic efficacy in several animal autoimmune disease models. Our study shows that short-term EPO therapy in MOG-immunized mice is associated with rapid expansion of the Treg cell population in peripheral lymphoid tissue and this EPO-induced expansion of Treg cells was sustained over long term follow up. In fact, EPO-treated EAE animals that manifested delayed onset of clinical disease and less neurologic damage were found to have higher numbers of Treg cells within both DILNs and spinal cord. The EPO-induced higher Treg population may play an important role in reducing the peripheral immune response to MOG autoantigen, and may be the major source of Tregs migrating into CNS to minimize inflammation within CNS white matter. A recent study showed that anti-inflammatory vasoactive intestinal peptide also prevents neurologic progression in MOG-EAE by inducing Treg cell expansion in peripheral lymphoid tissue and the CNS [34].

The other important subpopulation of CD4 helper T cells recently recognized to be a critical player in inflammatory autoimmune disease pathogenesis is the IL-17 cell. IL-17 is a potent proinflammatory cytokine and overproduction of IL-17 has been associated with a variety of human autoimmune diseases and their corresponding animal models. The kinetics and organ distribution of Th17 cells in our MOG_35–55_ induced EAE mice was very similar to the Th17 cell kinetics following proteolipid protein 139–151 peptide induced EAE in SLJ/J mice [39]. Th17 cells, like the MOG_40–54_ specific T cells, reside mainly in the peripheral lymphatics during the EAE induction phase after immunization. Later, when increased numbers of infiltrating inflammatory cells invade the CNS (12–20 days post MOG-immunization), Th17 cells, together with MOG-specific T effector cells, were easily detected in diseased spinal cords. EPO treatment abrogates the proliferation of peripheral Th17 cells during both the early induction phase as well as at later stages of EAE, and EPO treatment inhibits Th17 cell formation within the spinal cord during both acute and convalescent phases of EAE.

It is unknown by what mechanism EPO suppresses expansion of the Th17 cell subset in EAE animals. Since IL-6 has been reported to promote differentiation of naïve CD4 T cells into Th17 cells in vitro, the level of IL-6 secretion by inflammatory cells could be playing a crucial role for in vivo development and expansion of Th17 cells [40]. Our in vitro studies demonstrated significantly reduced IL-6 levels in EPO treated MOG-T cell cultures, suggesting that down-regulation of Th17 by EPO could be a consequence of reduced IL-6 secretion. A less severe inflammatory environment in peripheral lymphoid tissue as well as in the CNS after EPO therapy may prevent significant Th17 migration into the CNS, thereby delaying disease onset and inducing less severe CNS pathology.

The mechanism and precise signaling pathway by which EPO suppresses the immune response in the peripheral lymphoid tissues is also unknown. Since CD 11c expressing dendritic cells are pivotal in the control of developing immune responses, it may be that the EPO treatment induced massive 20-fold reduction in lymphoid dendritic cells coupled with marked down-regulation of MHC II expression on the surface of mononuclear cells in EAE is sufficient to block full development of the autoimmune process. This notion is supported by reports on 3 genetic conditions of mice where excessive DCs accumulate in the lymphoid tissues (DC-p35, CD11c-CRE Fas KI, and mice deficient in the immunoreceptor tyrosine-based inhibitor motif (Dcir)). These animals have greatly expanded dendritic cell populations; show exaggerated immune responses and subsequently develop systemic autoimmunity [41], [42], [43]. The classic EPO signaling pathway for erythropoiesis occurs through EPO ligand binding to EPO receptors and subsequent receptor dimerization, however there are recent reports claiming that EPO tissue protection is mediated by a different signaling pathway that involves binding to a hetero receptor composed of EPO receptor and the common beta receptor (βcR). The common beta receptor is used for IL-5, IL-3, and macrophage colony stimulating factor signaling and is upregulated during inflammatory responses [44] .

Beneficial therapeutic effects of exogenously administered whole molecule EPO have been described in several animal models of neurologic injury, including occlusive cerebral vascular disease, acute brain trauma, epilepsy, as well as in both autoimmune arthritis and EAE [14], [45], [46]. However, application of even medium-term EPO therapy for most acute conditions in humans remains improbable because EPO therapy may overly stimulate erythropoiesis with the subsequent risk of thromboembolic complications. To overcome this concern, whole molecule EPO administration would have to be limited to very short term use.

Other EPO molecular preparations, such as an asialo-form of EPO, carbamylated EPO (CEPO), or certain EPO mutants, have been reported to have protective effects in animals following experimental traumatic spinal cord injury or acute stroke without provoking an increase in red cell mass [47], [48], [49]. We have synthesized a library of low molecular weight cyclic EPO peptides that have been tested on the EAE animal model and we have identified a small domain that blocks paralysis in the acute EAE animal yet is devoid of hematopoietic effects [50]. This small domain appears to code for a region that contains most of the immuno/inflammatory modulating effects described in this report. Our evidence suggests that distinct functional and structural domain(s) co-exist within the whole molecule EPO, that retain tissue protective effects without manifesting hematopoietic side effects.

One of the surprising newly recognized consequences of EPO therapy in humans that has emerged over the past several years has been the unexpected negative effect of EPO therapy on survival in cancer patients [51]. A large clinical trial, the breast cancer erythropoietin survival trial (BEST) was terminated early because of an increased number of patients experiencing early demise in the EPO arm [52]. Poor survival was also reported in a randomized placebo controlled trial of Epoetin beta for the treatment of patients with head and neck cancer receiving radiotherapy [53]. The reason for the decreased cancer patient survival in these studies is unknown and enhanced tumor growth induced by signaling to EPO receptors growing on malignant cell surfaces has been suggested as one possible mechanism. The new observations described here in our report suggests another intriguing possibility that remains to be studied - could the immunomodulatory effects of EPO be contributing to suppressing immune function in the cancer patient?

In conclusion, we investigated the mechanism behind the neuroprotective effects of EPO in classic autoimmune EAE animals and found that EPO has striking pleiotropic immunomodulatory effects on the peripheral lymphatic tissues as well as on the CNS target organ. EPO appears to restrain the overwhelming autoimmune response in MOG-immunized EAE animals and restores it to a more balanced immune status. Our studies suggest that multiple alterations including a reduction in total autoreactive T cells, DCs, MHC expression as well as an alteration in the balance between Th17 cells and expanded Treg cell populations within peripheral lymphoid tissue and CNS all contribute to EPO-mediated neuroprotection.

## Materials and Methods

### Mice

C57BL/6 (8–10 weeks old) female mice were purchased from Charles River Laboratories and maintained in a conventional facility. The studies were conducted in accordance with the Animal Component of Research Protocol guidelines at the VA Hospital, East Orange, NJ, US.

### Animal Model of EAE

C57BL/6 mice were immunized subcutaneously at the tail base with 100 µl (200 µg) of MOG peptide_35–55_ dissolved in distilled water and emulsified with an equal volume of complete Freund's adjuvant supplemented with 4 mg/ml mycobacterium tuberculosis H37Ra [14]. Immediately after immunization, animals received an intravenous injection of 200 ng of bordetella pertussis toxin (List Biological Laboratories, Campbell, CA) in 200 µl PBS. Animals were weighed daily and assessed for clinical signs of EAE by two independent observers. We used the same clinical EAE scoring system to assess neurological deficit in our mouse EAE model as previously described [14] .

### Treatment protocol

EPO (Epoetin Alfa, Ortho Biotech products, LP) in 2,000 U/ml vial stock was stored at 4°C. Administration of EPO was initiated either at the time of immunization or delayed until day 7 post-immunization. Animals were treated with intravenous EPO (5000 U/kg/day×3 days followed by 1000 U/kg/day×3 days) in freshly prepared saline or sham treated for 6 days with an equal volume of saline.

### Mononuclear cell suspensions prepared from peripheral lymphoid and CNS tissue

At various time-points (before disease onset, at peak disease, and during recovery or later phases), animals were sacrificed, their draining inguinal lymph nodes and spleen removed for erythrocyte-depleted single-cell suspension preparations. The total MNCs were quantified subsequently. Mononuclear cells from the spinal cord were isolated following a published protocol by Korn T et al [54]. In brief, after perfusion through the left ventricle with cold PBS the pooled spinal cords from 6 animals were collected from MOG-immunized mice at each time-point. The spinal cords were cut into small pieces, minced, and passed through a tissue strainer and the mononuclear infiltrate obtained by purification through a Percoll gradient [54].

### Antibodies for flow cytometry

Cell surface staining of mouse single cell suspensions was obtained with mAbs directed against CD3, CD4, CD8, MHC class I, II and CD11c (BD PharMingen, San Diego, CA). Flow cytometry was performed on a BD FACScan machine (BD Biosciences). For intracellular IL-17 or Foxp3 staining, cells were first reacted with FITC-anti-CD4 or FITC-anti-CD4/APC-anti-CD25 (eBioscience, San Diego), then washed, fixed and permeabilized following the manufacturer's recommendations. The PE conjugated anti-Foxp3 or anti-IL-17 Mab was subsequently added. The samples were subjected to flow cytometry after multiple washings. Flow cytometry data was analyzed using CellQuest software and the Flowjo analysis program.

### EPO effects on MOG-EAE-specific T cells in tissue culture

MNCs were obtained from EAE mice spleens 5–7 days post MOG_35–55_ immunization. T cells were isolated from the single cell suspensions using MAC pan-T cell beads [55]. The MOG-specific T cells were then enriched by repeated stimulation with syngeneic splenic antigen presenting cells pulsed with MOG_35–55_ peptide antigen. To determine the biological effect of EPO on antigen specific T cells and their secreted cytokines, the in vitro enriched MOG-specific T cells were washed three times with cold PBS and then incubated with log dilutions of EPO (1, 10 and 100 U/ml) in culture medium (RPMI1640 supplemented with 10% FCS) for 48–72 hrs. Control cells were incubated with medium alone. MOG_44–54_/H2-D^b^ and control NP_366–374_/H2-D^b^ dimers were freshly prepared by adding the truncated peptides to a recombinant soluble dimeric mouse H-2D^b^:IgG1 fusion protein (BD Pharmingen, San Diego, CA). Cell culture supernatants were collected from each experimental sample well for cytokine profile analysis (below). Flow cytometry of either EPO treated cells or control cells was performed using dual-color staining with peptide dimer complexes and anti-CD3 mAb to determine the level of MOG specific effector T cells [19].

### Effect of EPO on cytokine levels in MOG-EAE specific T cell cultures

Luminex technology was used to detect multiple cytokines and chemokines simultaneously within the pre-treated EAE T cell culture supernatants (above). Commercial kits (Upstate, Lake Placid, NY) containing multi-cytokine beads (IL-1β, IL-2, IL-4, IL-5, IL-6, IL-10, GM-CSF, IL-12, TNFα and IFN-γ) were run with their enclosed buffers and standards, following directions from the manufacturer. To obtain concentration values, mean fluorescence intensities from the bead combinations were analyzed by the MasterPlex QT quantification software.

### Immunohistochemistry

Spinal cords were obtained from sham or EPO treated MOG-EAE animals. Immunohistochemical staining for intracellular Foxp3 was performed using biotinylated Foxp3 antibody (eBioscience, San Diego, CA) and an avidin-biotin immunoperoxidase detection system on paraffin-embedded spinal cord sections.

### Statistical analysis

Data are presented as the mean±SEM. The Mann-Whitney U test was used to determine the significance of the intergroup difference in EAE clinical scores and duration of maximum neurological deficit. The composite data was analyzed by the Kruskal-Wallis one-way analysis of variance. Values of p<0.05 were considered significant.
